# Supplementary material for: LC-MS/MS Method for the Quantification of PARP Inhibitors Olaparib, Rucaparib and Niraparib in Human Plasma and Dried Blood Spot: Development, Validation and Clinical Validation for Therapeutic Drug Monitoring
Source: Pharmaceutics. 2023 May 18;15(5):1524. doi: 10.3390/pharmaceutics15051524 (PMC10221204; doi:10.3390/pharmaceutics15051524)
Supplement: Supplementary file 1 [file pharmaceutics-15-01524-s001.zip › Informed consent_CLEAN.pdf]

Dr. Giuseppe Toffoli, Direttore Struttura Operativa Complessa di Farmacologia Sperimentale e Clinica
Dipartimento di Ricerca Traslazionale Oncologia Molecolare, Diagnostica Avanzata e Terapie Cellulari
☎ +39.0434.659612, Fax +39.0434.659799
✉ gtoffoli@cro.it

MODULO PER IL CONSENSO INFORMATO

INFORMAZIONI GENERALI

Titolo dello studio: Farmacogenetica, Monitoraggio Terapeutico dei Farmaci (TDM) e Farmacovigilanza attiva quali strumenti innovativi finalizzati all'ottimizzazione/appropriatezza della terapia farmacologica e alla minimizzazione dei rischi di ADRs nella pratica clinica: un approccio multidisciplinare esportabile a livello nazionale
Codice del protocollo: CRO-2022-14
Promotore dello studio: Centro Regionale di Farmacovigilanza- Servizio pianificazione, controllo, economia sanitaria e assistenza farmaceutica- Direzione Centrale Salute, politiche sociali e disabilità- regione Autonoma Friuli-Venezia Giulia
Sperimentatore principale: Dr. Giuseppe Toffoli, Direttore della SOC di Farmacologia Sperimentale e Clinica del Centro di Riferimento Oncologico, Istituto Nazionale Tumori, IRCCS, di Aviano (Italy)
tel. +39-0434-659612 (659667)
FAX +39-0434-659659
email: gtoffoli@cro.it

Io sottoscritto ________________________________, nato a __________________________________ il ________________________, dichiaro:

- di aver ricevuto dal Dr. Toffoli Giuseppe, o chi per lui, informazioni scritte (attraverso l'informativa Versione 1.0 del 16/12/2021) e verbali sullo studio in oggetto, compresi rischi e benefici connessi alla sperimentazione;
- di aver avuto la possibilità di porre domande sullo studio;
- di aver avuto il tempo necessario prima di decidere liberamente se partecipare o meno;
- di non aver avuto coercizioni alla partecipazione allo studio e di aver ricevuto l'informazione della possibilità di poterne uscire in qualsiasi momento;

Ai sensi del codice in materia di protezione dei dati personali Il trattamento dei dati personali ha come basi giuridiche di riferimento il D.Lgs. n. 196/2003 per la tutela della privacy e successive autorizzazioni al trattamento dei dati genetici e il Regolamento UE 2016/679 (GDPR) e successive disposizioni per l'adeguamento emanate dal Garante per la protezione dei dati personali dichiaro:

- Di volere ☐ / non volere ☐ partecipare allo studio di Farmacovigilanza attiva;
- Di volere ☐ / non volere ☐ essere informato dal medico sui risultati della ricerca;
- Di volere ☐ / non volere ☐ informare il Medico di Medicina Generale della partecipazione allo studio.

Poiché lo studio prevede la raccolta di campioni biologici legati all'attività diagnostica:

- Acconsento ☐ / non acconsento ☐ alla conservazione dei miei campioni biologici secondo le modalità descritte dall'informativa che ho letto.
- Acconsento ☐ /non acconsento ☐ all'esecuzione di analisi future in seguito ad anonimizzazione dei campioni secondo le modalità descritte dall'informativa che ho letto.

Poiché lo studio prevede la partecipazione al sub-studio di fattibilità del campionamento del sangue per TDM attraverso la tecnica del dried blood spot (DBS):

- ☐ Desidero / ☐ Non desidero partecipare al sub-studio di fattibilità dell'utilizzo di DBS.

Dr. Giuseppe Toffoli, Direttore Struttura Operativa Complessa di Farmacologia Sperimentale e Clinica
Dipartimento di Ricerca Traslazionale Oncologia Molecolare, Diagnostica Avanzata e Terapie Cellulari
☎ +39.0434.659612, Fax +39.0434.659799
✉ gtoffoli@cro.it

Firmando questo modulo di consenso informato non ho rinunciato ad alcun diritto legale di cui potrei altrimenti godere come partecipante a uno studio di ricerca. Autorizzo la raccolta, l'uso e la divulgazione delle informazioni mediche che mi riguardano, secondo quanto specificato nel foglio informativo (versione 1.0 del 16/12/2021).
Pertanto, do liberamente il mio consenso a prendere parte allo studio di farmacovigilanza attiva.

(La firma su questo modulo non verrà ad incidere sui miei diritti)
Nome e cognome per esteso in stampatello del/la PAZIENTE se sottoscrive IN PROPRIO ovvero del GENITORE/ ALTRO ESERCENTE POTESTÀ GENITORIALE / RAPPRESENTANTE LEGALE DI PERSONA INTERDETTA O INABILITATA / TUTORE / AMMINISTRATORE DI SOSTEGNO.

____________________ ______________________
NOME COGNOME

Data: ________________ Firma ____
(leggibile)
PAZIENTE IN PROPRIO ovvero GENITORE 1/ ALTRO ESERCENTE POTESTÀ GENITORIALE / RAPPRESENTANTE LEGALE DI PERSONA INTERDETTA O INABILITATA / TUTORE / AMMINISTRATORE DI SOSTEGNO

Data: ________________ (eventuale seconda) Firma ________________________________
(leggibile)

***

Se il presente modulo di consenso informato viene letto al/alla partecipante allo studio in quanto non è in grado di leggerlo e/o firmarlo autonomamente, due testimoni imparziali, indipendenti dallo studio, devono essere presenti al momento della firma del modulo di consenso e devono rilasciare idonea dichiarazione:

FIRMA DEI TESTIMONI IMPARZIALI (*compilare solo se ricorre la necessità*)

(Qualora il paziente non fosse in grado di leggere e/o scrivere o di fornire per iscritto il consenso informato – D.L. n. 211 del 24-06-03, articolo 2, comma 1, lettera l.)

Il testimone deve saper leggere e scrivere, essere una persona che sia indipendente dallo studio, non essere dipendente del Promotore dello studio, non essere coniuge o parente di primo grado del paziente e non essere coinvolto nello studio in alcun ruolo e non deve poter essere influenzato in alcun modo dalle persone coinvolte nello studio. Il testimone deve partecipare alla procedura di informazione del paziente leggendo il consenso informato e ogni altra informazione scritta fornita al paziente partecipante).

Se due Testimoni imparziali indipendenti prestano il consenso per conto del/della paziente, il/la paziente dovrà firmare il consenso informato non appena sarà in grado di farlo.

Io sottoscritto dichiaro che al/alla paziente sono state correttamente spiegate le modalità di trattamento dei suoi dati personali e che il/la paziente ha acconsentito liberamente al trattamento dei suoi dati personali per gli scopi della ricerca nei limiti e con le modalità indicate nell'informativa fornita con il presente documento.

Nome e cognome per esteso del testimone imparziale 1 (in stampatello): ______________________________

Data: ____________________ Firma del testimone imparziale 1: ______________________________
(leggibile)

Dr. Giuseppe Toffoli, Direttore Struttura Operativa Complessa di Farmacologia Sperimentale e Clinica
Dipartimento di Ricerca Traslazionale Oncologia Molecolare, Diagnostica Avanzata e Terapie Cellulari
☎ +39.0434.659612, Fax +39.0434.659799
✉ gtoffoli@cro.it

Io sottoscritto dichiaro che al/alla paziente sono state correttamente spiegate le modalità di trattamento dei suoi dati personali e che il/la paziente ha acconsentito liberamente al trattamento dei suoi dati personali per gli scopi della ricerca nei limiti e con le modalità indicate nell'informativa fornita con il presente documento.

Nome e cognome per esteso del testimone imparziale 2 (in stampatello): ____________________________

Data: _____________________ Firma del testimone imparziale 2: ________________________________
(leggibile)

DICHIARAZIONE DI AVVENUTA INFORMAZIONE DEL MEDICO CHE HA PRESENTATO L'INFORMATIVA AL/ALLA PAZIENTE, AL RAPPRESENTANTE LEGALE DI PERSONA INTERDETTA O INABILITATA / TUTORE / AMMINISTRATORE DI SOSTEGNO ED AGLI EVENTUALI TESTIMONI IMPARZIALI

Io sottoscritto/a (nome e cognome dello Sperimentatore informante in stampatello) __________________ ________________________________ dichiaro che al/alla paziente ovvero al rappresentante legale di persona interdetta o inabilitata / tutore / amministratore di sostegno sono stati correttamente spiegate le modalità di trattamento dei dati personali e che il/la paziente ovvero il rappresentante legale di persona interdetta o inabilitata / tutore / amministratore di sostegno ha/hanno acconsentito liberamente all'utilizzo dei propri dati personali per gli scopi della ricerca nei limiti e con le modalità indicate nell'informativa fornita con il presente documento.

Ho constatato che il/la paziente è in grado di leggere e comprendere il presente modulo redatto in lingua italiana, che ha compreso tutte le informazioni in esso contenute e che è stato/a informato/a in modo esaustivo su trattamento dei dati personali ai fini del presente studio, così come sulla natura e sulla revocabilità del suo consenso al trattamento dei suoi dati personali.

Dichiaro altresì di aver fornito copia della presente informativa e di aver ottenuto il consenso del/la paziente ovvero dal rappresentante legale di persona interdetta o inabilitata / tutore / amministratore di sostegno e degli eventuali testimoni imparziali.

NOME e COGNOME dello Sperimentatore informante: ____
(in stampatello)

Data: ___________ Firma dello Sperimentatore informante: _______________________________________
(leggibile)

REDATTO IN DUE COPIE, L'ORIGINALE DA CONSERVARSI A CURA DEL MEDICO DELLO STUDIO, E LA COPIA DA CONSEGNARE AL/ALLA PAZIENTE, ALL'EVENTUALE GENITORE / ALTRO ESERCENTE POTESTÀ GENITORIALE / RAPPRESENTANTE LEGALE DI PERSONA INTERDETTA O INABILITATA / TUTORE / AMMINISTRATORE DI SOSTEGNO ED AGLI EVENTUALI TESTIMONI IMPARZIALI.

Dr. Giuseppe Toffoli, Direttore Struttura Operativa Complessa di Farmacologia Sperimentale e Clinica
Dipartimento di Ricerca Traslazionale Oncologia Molecolare, Diagnostica Avanzata e Terapie Cellulari
☎ +39.0434.659612, Fax +39.0434.659799
✉ gtoffoli@cro.it

MODULO PER LA REVOCA DEL CONSENSO

INFORMAZIONI GENERALI

Titolo dello studio: Farmacogenetica, Monitoraggio Terapeutico dei Farmaci (TDM) e Farmacovigilanza attiva quali strumenti innovativi finalizzati all'ottimizzazione/appropriatezza della terapia farmacologica e alla minimizzazione dei rischi di ADRs nella pratica clinica: un approccio multidisciplinare esportabile a livello nazionale
Codice del protocollo: CRO-2022-14
Promotore dello studio: Centro Regionale di Farmacovigilanza- Servizio pianificazione, controllo, economia sanitaria e assistenza farmaceutica- Direzione Centrale Salute, politiche sociali e disabilità- regione Autonoma Friuli-Venezia Giulia
Sperimentatore principale: Dr. Giuseppe Toffoli, Direttore della SOC di Farmacologia Sperimentale e Clinica del Centro di Riferimento Oncologico, Istituto Nazionale Tumori, IRCCS, di Aviano (Italy)
tel. +39-0434-659612 (659667)
FAX +39-0434-659659
email: gtoffoli@cro.it

Io sottoscritto/a (*Nome e Cognome in stampatello*) ____________________ ________________________,

nato/a __________________ il: __/__/_____

residente in ______________________________ Prov. __________ CAP ___________

Via _____________________n° _____Tel. ____________________

in proprio, in quanto soggetto maggiorenne con piena capacità d'agire e di intendere e volere,

ovvero nella qualità di

☐ TUTORE (per soggetto INTERDETTO)

☐ AMMINISTRATORE DI SOSTEGNO, tenuto conto della volontà del beneficiario, in relazione al grado di intendere e volere del soggetto cui è stato nominato l'amministratore di sostegno

☐ RAPPRESENTANTE LEGALE DI PERSONA INTERDETTA O INABILITATA

Di: (*Nome e Cognome in stampatello del* ***partecipante allo studio/ricerca***) ____________________

____,

nato/a a ____ il ______________________________
residente in ____ Prov. __________ CAP ___________

Via _____________________n° _____Tel. ____________________

Dr. Giuseppe Toffoli, Direttore Struttura Operativa Complessa di Farmacologia Sperimentale e Clinica
Dipartimento di Ricerca Traslazionale Oncologia Molecolare, Diagnostica Avanzata e Terapie Cellulari
☎ +39.0434.659612, Fax +39.0434.659799
✉ gtoffoli@cro.it

Con riferimento al consenso al trattamento dei dati personali e sanitari necessari allo svolgimento dello studio per gli scopi della ricerca, nei limiti e con le modalità indicate nell'informativa da me precedentemente sottoscritta,

DICHIARO DI VOLER REVOCARE LIBERAMENTE IL MIO CONSENSO,

senza pregiudicare le mie cure mediche né i miei diritti legali e senza che tale scelta modifichi in alcun modo i miei rapporti con il personale e la struttura sanitaria.

Sono consapevole, sottoscrivendo questo modulo, che da questo momento in poi non verranno più raccolti ulteriori dati.

Sono altresì a conoscenza del fatto che i dati raccolti fino al momento del mio ritiro rimangono parte dei risultati dello studio, pertanto le informazioni personali già ottenute saranno conservate per assicurare la corretta valutazione dei risultati dello studio e la conformità alle disposizioni di legge.

☐ ACCONSENTO ☐ NON ACCONSENTO

all'utilizzazione dei dati già raccolti per determinare, senza alterarli, i risultati della ricerca, o per l'accertamento, l'esercizio o la difesa di un diritto in sede giudiziaria.

Nome e cognome per esteso in stampatello del/la PAZIENTE se sottoscrive IN PROPRIO ovvero del RAPPRESENTANTE LEGALE DI PERSONA INTERDETTA O INABILITATA / TUTORE / AMMINISTRATORE DI SOSTEGNO

Nome e cognome per esteso del paziente ovvero del rappresentante legale (in stampatello): ____________

Data: ________________ Firma ____
(leggibile)

***

Se il presente modulo di revoca viene letto al/alla partecipante allo studio in quanto non è in grado di leggerlo e/o firmarlo autonomamente, due testimoni imparziali, indipendenti dallo studio, devono essere presenti al momento della firma del modulo di consenso e devono rilasciare idonea dichiarazione:

FIRMA DEI TESTIMONI IMPARZIALI (*compilare solo se ricorre la necessità*)

(Qualora il paziente non fosse in grado di leggere e/o scrivere o di fornire per iscritto la revoca al consenso informato – D.L. n. 211 del 24-06-03, articolo 2, comma 1, lettera l.)

Il testimone deve saper leggere e scrivere, essere una persona che sia indipendente dallo studio, non essere dipendente del Promotore dello studio, non essere coniuge o parente di primo grado del paziente e non essere coinvolto nello studio in alcun ruolo e non deve poter essere influenzato in alcun modo dalle persone coinvolte nello studio. Il testimone deve partecipare alla procedura di informazione del paziente leggendo il consenso informato e ogni altra informazione scritta fornita al paziente partecipante).

Se due Testimoni imparziali indipendenti revocano il consenso per conto del/della paziente, il/la paziente dovrà firmare il consenso informato non appena sarà in grado di farlo.

Io sottoscritto dichiaro che al/alla paziente è stato correttamente spiegato cosa comporta la revoca del consenso al trattamento dei dati personali dell'interessato partecipante allo studio, e che il/la paziente ovvero il rappresentante legale di persona interdetta o inabilitata / tutore / amministratore di sostegno hanno liberamente e volontariamente revocato il consenso dato in precedenza.

Nome e cognome per esteso del testimone imparziale 1 (in stampatello): ___________________________

Dr. Giuseppe Toffoli, Direttore Struttura Operativa Complessa di Farmacologia Sperimentale e Clinica
Dipartimento di Ricerca Traslazionale Oncologia Molecolare, Diagnostica Avanzata e Terapie Cellulari
☎ +39.0434.659612, Fax +39.0434.659799
✉ gtoffoli@cro.it

Data: ____________________ Firma del testimone imparziale 1: ______________________________
(leggibile)

Nome e cognome per esteso del testimone imparziale 2 (in stampatello): ___________________________

Data: ____________________ Firma del testimone imparziale 2: _______________________________
(leggibile)

DICHIARAZIONE DI AVVENUTA INFORMAZIONE DEL MEDICO CHE HA RACCOLTO LA REVOCA AL CONSENSO AL TRATATMENTO DEI DATI PERSONALI DAL/DALLA PAZIENTEOVVERO DAL RAPPRESENTANTE LEGALE DI PERSONA INTERDETTA O INABILITATA / TUTORE / AMMINISTRATORE DI SOSTEGNO ED AGLI EVENTUALI TESTIMONI IMPARZIALI

Io sottoscritto/a (nome e cognome dello Sperimentatore informante in stampatello) __________________ _____________________ dichiaro che al/alla paziente ovvero al rappresentante legale di persona interdetta o inabilitata / tutore / amministratore di sostegno sono state correttamente spiegate le modalità di trattamento dei dati personali e che il/la paziente ovvero il rappresentante legale di persona interdetta o inabilitata / tutore / amministratore di sostegno ha/hanno revocato liberamente e volontariamente il consenso precedentemente rilasciato all'utilizzo dei dati personali per gli scopi della ricerca nei limiti e con le modalità indicate nell'informativa fornita con il presente documento.

Ho constatato che il/la paziente è in grado di leggere e comprendere il presente modulo redatto in lingua italiana, che ha compreso tutte le informazioni in esso contenute e che è stato/a informato/a in modo esaustivo sul significato della revoca al consenso al trattamento dei dati personali rilasciato in precedenza.

Dichiaro altresì di aver fornito copia della presente revoca al/agli interessato/i.

NOME e COGNOME dello Sperimentatore informante: ____
(in stampatello)

Data: ___________ Firma dello Sperimentatore informante: _____________________________________
(leggibile)

REDATTO IN DUE COPIE, L'ORIGINALE DA CONSERVARSI A CURA DEL MEDICO DELLO STUDIO, E LA COPIA DA CONSEGNARE AL/ALLA PAZIENTE, ALL'EVENTUALE GENITORE / ALTRO ESERCENTE POTESTÀ GENITORIALE / RAPPRESENTANTE LEGALE DI PERSONA INTERDETTA O INABILITATA / TUTORE / AMMINISTRATORE DI SOSTEGNO ED AGLI EVENTUALI TESTIMONI IMPARZIALI.
