# Supplementary material for: LC-MS/MS Method for the Quantification of PARP Inhibitors Olaparib, Rucaparib and Niraparib in Human Plasma and Dried Blood Spot: Development, Validation and Clinical Validation for Therapeutic Drug Monitoring
Source: Pharmaceutics. 2023 May 18;15(5):1524. doi: 10.3390/pharmaceutics15051524 (PMC10221204; doi:10.3390/pharmaceutics15051524)
Supplement: Supplementary file 1 [file pharmaceutics-15-01524-s001.zip › Supplementary material.pdf]

Supplementary material

Table S1. General (source and gas) mass spectrometric parameters.

Ion Source	Turbo Spray
Polarity	Positive ionisation mode
Curtain gas (CUR)	35 a.u.
Collision gas (CAD)	3 a.u.
IonSpray Voltage (IS)	1500 V
Temperature (TEM)	450 °C
Ion Source Gas 1 (GS1)	40 a.u.
Ion Source Gas 2 (GS2)	50 a.u.

a.u.: arbitrary units.

Table S2. Analytes specific mass spectrometric parameters, listed in the exact acquisition order.

Analyte	Precursor ion (*m/z*)	Product ion (*m/z*)	DP (V)	EP (V)	CE (V)	CXP (V)	Dwell time (msec)
Rucaparib	324	293	50	10	15	20	70
	324	264	50	10	35	20	10
Olaparib	435	367	100	10	29	25	70
	435	281	100	10	45	20	10
Niraparib	321	304	75	10	30	22	70
	321	205	75	10	45	15	10
Rucaparib IS	328	285	50	10	28	20	30
Olaparib IS	443	375	100	10	30	25	30
Niraparib IS	327	310	80	10	30	23	30

DP: declustering potential, EP: entrance potential, CE: collision energy, CXP: collision-cell exit potential.

Table S3. Evaluation of the best weighting factor (w) for the linear regression analysis.

Analyte	w	SS	r	AIC	∑%RE
Rucaparib	1	86.08	0.8952	66.74	284.547
	1/x	18.52	0.9385	-85.58	137.06
	$1/x^2$	1.12	0.9253	-272.03	120.089
Niraparib	1	70.71	0.9859	-98.97	298.14
	1/x	15.43	0.9920	-249.74	135.85
	$1/x^2$	0.96	0.9895	-429.44	112.02
Olaparib	1	79.32	0.9748	-47.98	242.18
	1/x	17.11	0.9860	-201.44	109.67
	$1/x^2$	1.05	0.9827	-386.88	92.40

w: weighting factor ; SS: sum of squares; r: correlation coefficient; AIC: Akaike's Information Criterion; ∑%RE: sum of the absolute %RE.

Table S4. Precision and accuracy data of calibration curves for both plasma and DBS matrices.

Analyte	Nominal	Plasma (n=3)	DBS (n=3)

	concentration (ng/mL)	Mean concentration (ng/mL)	CV%	Acc%	Mean concentration (ng/mL)	CV%	Acc%
Rucaparib r≥0.9970 (plasma); r≥0.9988 (DBS)	100	100	6	100	98	3	98
	200	198	7	99	207	4	104
	500	502	6	100	500	3	100
	1000	987	6	99	989	3	99
	2000	1988	5	99	2012	3	101
	3000	3001	5	100	3013	4	100
	4000	4066	6	102	4004	3	100
	5000	5015	5	100	4906	3	98
Niraparib r≥0.9993 (plasma); r≥0.9994 (DBS)	60	60	2	99	59	2	99
	120	121	3	101	122	3	101
	300	307	2	102	307	2	102
	600	604	5	101	596	3	99
	1200	1186	2	99	1190	2	99
	1800	1807	2	100	1774	2	99
	2400	2396	4	100	2426	2	101
	3000	2944	2	98	2973	3	99
Olaparib r≥0.9985 (plasma); r≥0.9993 (DBS)	140	139	4	99	140	3	100
	280	285	4	102	281	2	100
	700	703	4	100	695	2	99
	1400	1396	4	100	1403	3	100
	2800	2795	4	100	2808	4	100
	4200	4130	2	98	4179	3	99
	5600	5580	3	100	5652	2	101
	7000	7081	5	101	6963	2	99

Acc%: percentage of accuracy; CV%: coefficient of variation.

Table S5. Dilution integrity data for the method in human plasma (n=5).

Analyte	Nominal concentration (ng/mL)	Mean concentration (ng/mL)	CV%	Acc%
Rucaparib	1414	1532	5	108
Niraparib	888	920	5	104
Olaparib	1957	2009	1	103

Acc%: percentage of accuracy; CV%: coefficient of variation.

Table S6. Hematocrit effect (25% and 55% of Hct) on accuracy and precision.

Analyte	Nominal concentration (ng/mL)	25% Hct (n=3)			55% Hct (n=3)		
		Mean concentration (ng/mL)	CV%	Acc%	Mean concentration (ng/mL)	CV%	Acc%
Rucaparib	100	121	1	121	91	4	91
	255	314	4	123	201	31	79
	1700	1997	1	117	1601	5	94
	4250	5134	4	121	3702	5	87

Niraparib	60	69	3	114	52	4	87
	153	185	4	121	117	33	76
	1020	1235	3	121	916	7	90
	2460	2973	5	117	2165	3	85
Olaparib	140	148	3	106	133	1	95
	357	382	4	107	305	35	85
	2380	2552	1	107	2407	4	101
	5950	6299	2	106	5826	1	98

Acc%: percentage of accuracy; CV%: coefficient of variation; Hct: hematocrit.

Table S7. Clinical validation data for rucaparib.

Sample number	C_{pla} (ng/mL)	C_{DBS} (ng/mL)	EC_{pla} (ng/mL)
1	1270	1640*	1149
2	1069	1602	1123
3	1526	1957	1372
4	1773	2368	1660
5	2007	2676	1876
6	1526	2124	1489
7	1119	1808	1267
8	1049	1679	1177
9	1045	1736	1217
10	1701	3238	2269
11	1287	1946	1364
12	2116	2626	1840
13	802	937	657
14	1667	2388	1674
15	1082	1371	961
16	3338	4311	3022

* mean value from incurred sample reanalysis, obtained with a calibration curve with Hct=41

Table S8. Clinical validation data for niraparib.

Sample number	C_{pla} (ng/mL)	C_{DBS} (ng/mL)	EC_{pla} (ng/mL)
1	888	1276*	886
2	831	1215	844
3	868	1271	883
4	831	1471	1022
5	835	1528	1061
6	812	1065	739
7	172	267	185
8	157	227	157
9	188	272	189
10	227	305	212
11	253	342	237
12	175	235	163
13	355	440	306
14	304	403	279
15	281	403	280
16	310	500*	347
17	246	355	247

18	425	625	434
19	498	798	554
20	134	178	124
21	390	572	397
22	236	309	214
23	414	614	427
24	558	706	491
25	358	530	368
26	433	625	434
27	1151	1308	908
28	313	436	302
29	405	745	517
30	372	479	332
31	542	746	518
32	207	356	247
33	227	361	250
34	610	970	674
35	748	1091	757
36	244	356	247
37	313	376	261
38	308	415	288
39	707	831	577
40	329	504	350
41	567	804	559
42	547	769	534
43	403	538	374

* mean value from incurred sample reanalysis, obtained with a calibration curve with Hct=41

Table S9. Clinical validation data for olaparib.

Sample number	C_{pla} (ng/mL)	C_{DBS} (ng/mL)	EC_{pla} (ng/mL)
1	3326	2536*	3531
2	2651	1915	2666
3	5290	3893	5420
4	2244	1543	2149
5	406	284*	395
6	323	250	348
7	390	306	426
8	375	278	387
9	868	625	870
10	1442	1098	1530
11	632	526	732
12	738	477	664
13	1653	1153	1605
14	2261	1431	1993
15	1472	826	1150
16	1062	648	902
17	2529	1767	2461
18	1397	1001	1394
19	1902	1352	1883
20	2389	1746	2431
21	2251	1645	2290
22	361	259	361
23	785	568	791
24	583	445	620
25	809	554	771
26	1888	1329	1851
27	659	503	700
28	335	248	346
29	983	799	1113
30	3886	2951	4109
31	241	148	206

32	496	324	451
33	625	390	543
34	354	263	366
35	574	439	611
36	621	504	702
37	1196	812	1131
38	2489	1618	2254
39	1268	924	1287
40	1635	1201	1672
41	1576	1190	1657
42	590	461	642
43	2053	1473	2052
44	1541	1095	1524
45	1548	1090	1518
46	977	713	993
47	533	401	558
48	1993	1612	2245
49	811	564	786
50	1092	691	963
51	1299	898	1251
52	1260	927	1290

* mean value from incurred sample reanalysis, obtained with a calibration curve with Hct=41

Table S10. Incurred sample reanalysis (ISR) for plasma samples.

Analyte	1st analysis	2nd analysis	%diff
Rucaparib	653	654	0
	837	775	8
	878	872	-1
Niraparib	383	380	1
	694	696	0
	412	428	4
	554	551	-1
Olaparib	2694	2663	-1
	2851	2673	-6
	4355	4362	0
	1331	1307	-2

%diff: percentage of difference.

Table S11. Incurred sample reanalysis (ISR) for DBS samples.

Analyte	1st analysis	2nd analysis	%diff
Rucaparib	1610	1670	-4
	1808	1887	-4
	1679	1667	1
	1957	1950	0
Niraparib	1306	1247	5
	489	512	-5
	267	239	11
Olaparib	304	265	14
	853	861	-1
	2665	2407	10
	250	220	13

	4202	4235	-1
	799	783	2

%diff: percentage of difference.
