# Supplementary figures and images for: LC-MS/MS Method for the Quantification of PARP Inhibitors Olaparib, Rucaparib and Niraparib in Human Plasma and Dried Blood Spot: Development, Validation and Clinical Validation for Therapeutic Drug Monitoring
Source: Pharmaceutics. 2023 May 18;15(5):1524. doi: 10.3390/pharmaceutics15051524 (PMC10221204; doi:10.3390/pharmaceutics15051524)

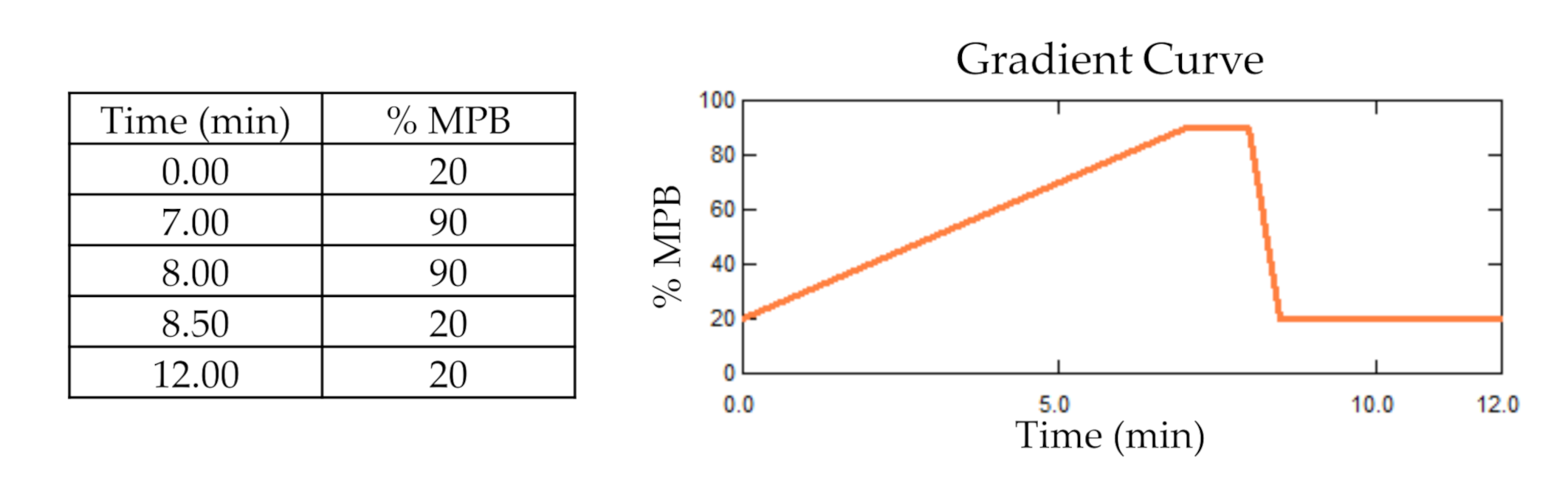

Supplement: Supplementary file 1 [file pharmaceutics-15-01524-s001.zip › Figure S1.tif]

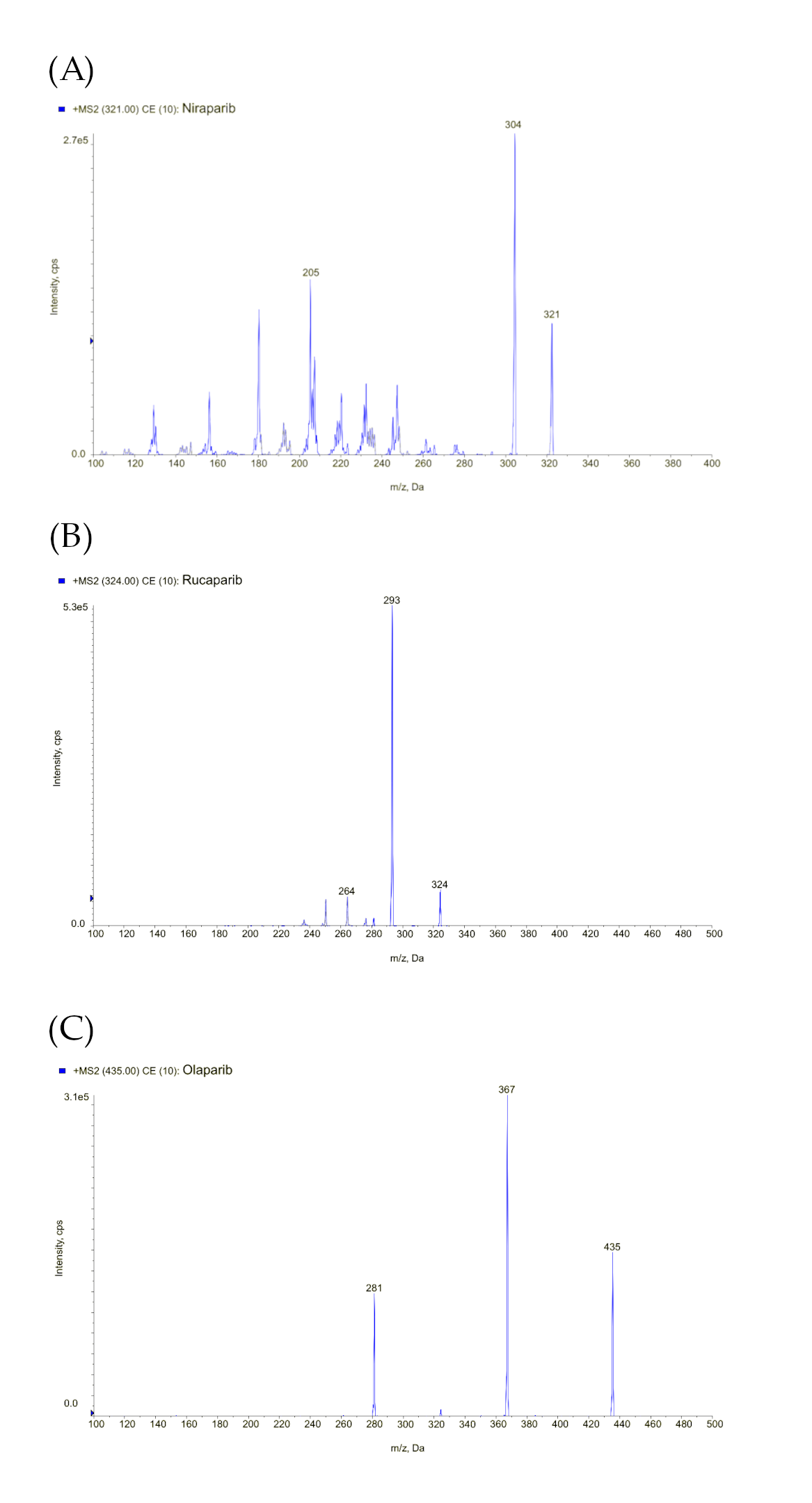

Supplement: Supplementary file 1 [file pharmaceutics-15-01524-s001.zip › Figure S2.tif]

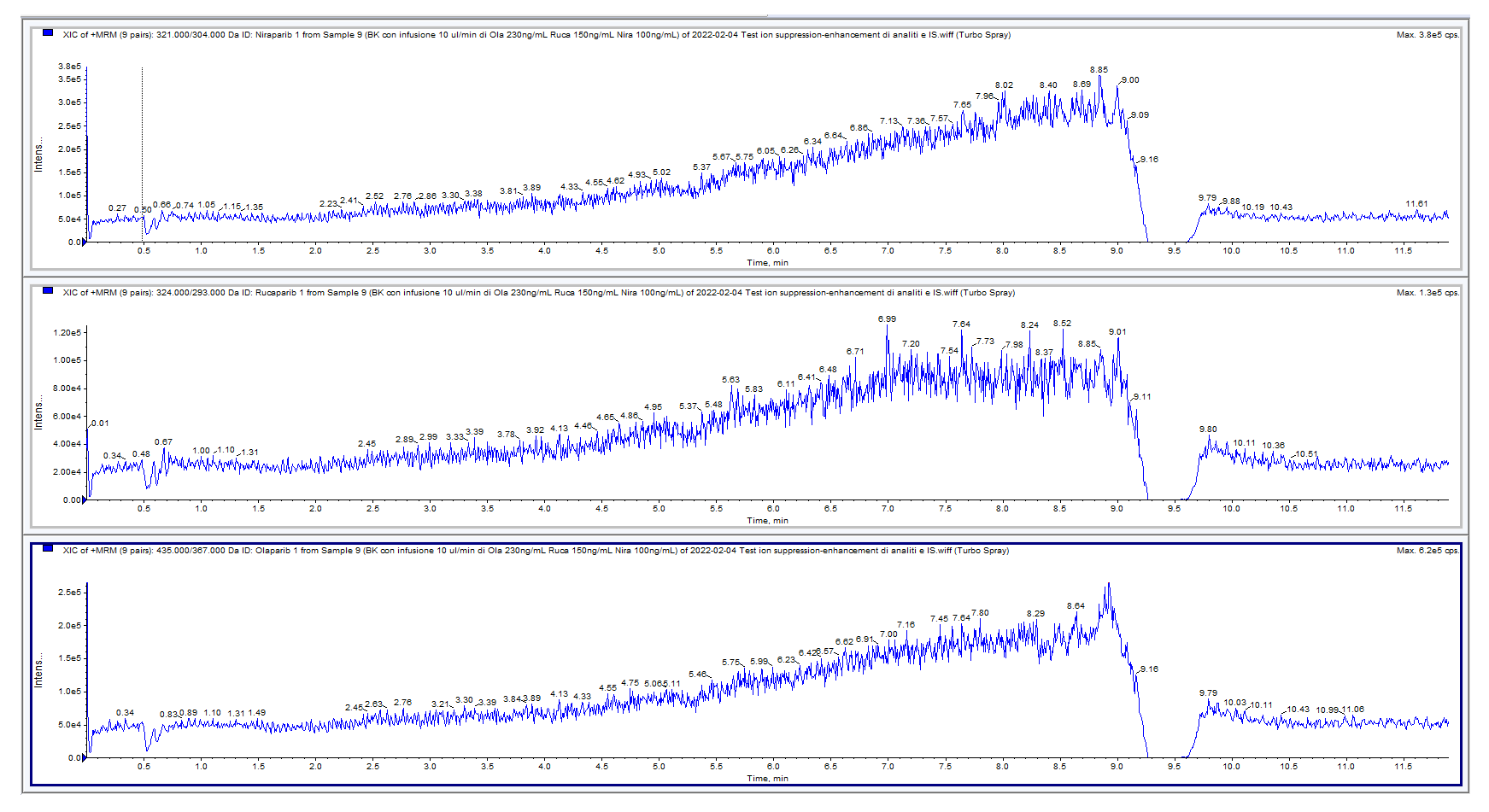

Supplement: Supplementary file 1 [file pharmaceutics-15-01524-s001.zip › Figure S3.tif]

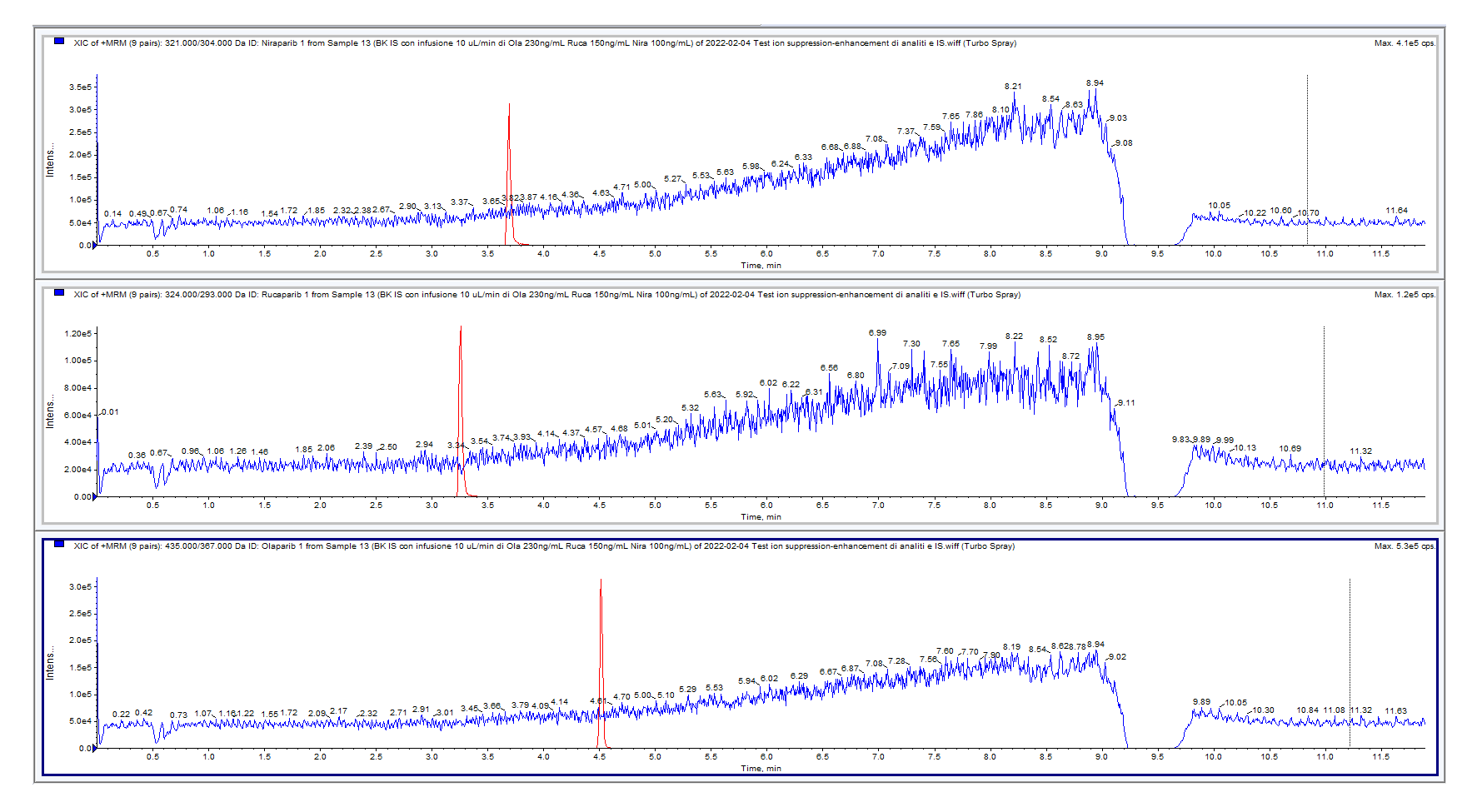

Supplement: Supplementary file 1 [file pharmaceutics-15-01524-s001.zip › Figure S4.tif]

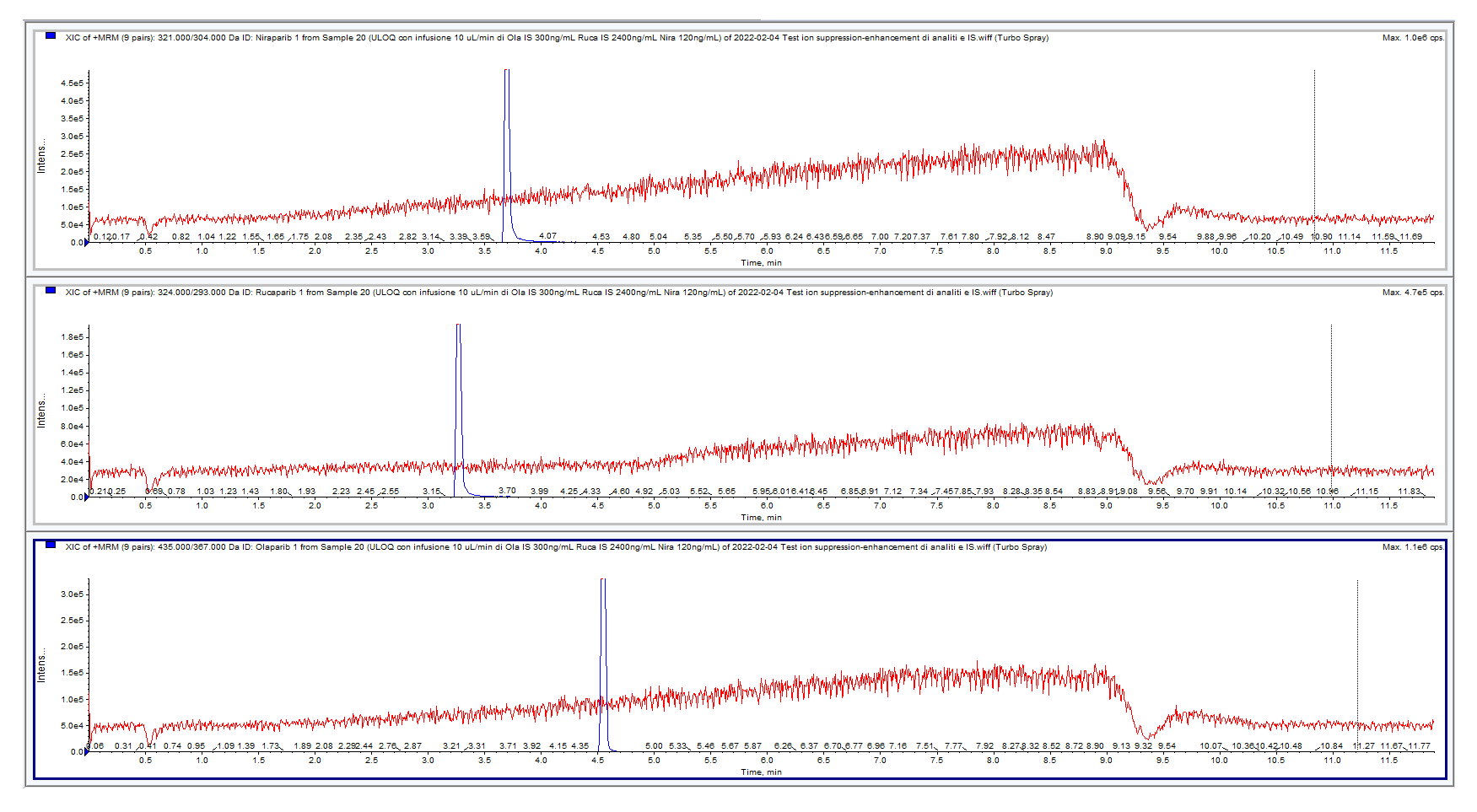

Supplement: Supplementary file 1 [file pharmaceutics-15-01524-s001.zip › Figure S5.tif]

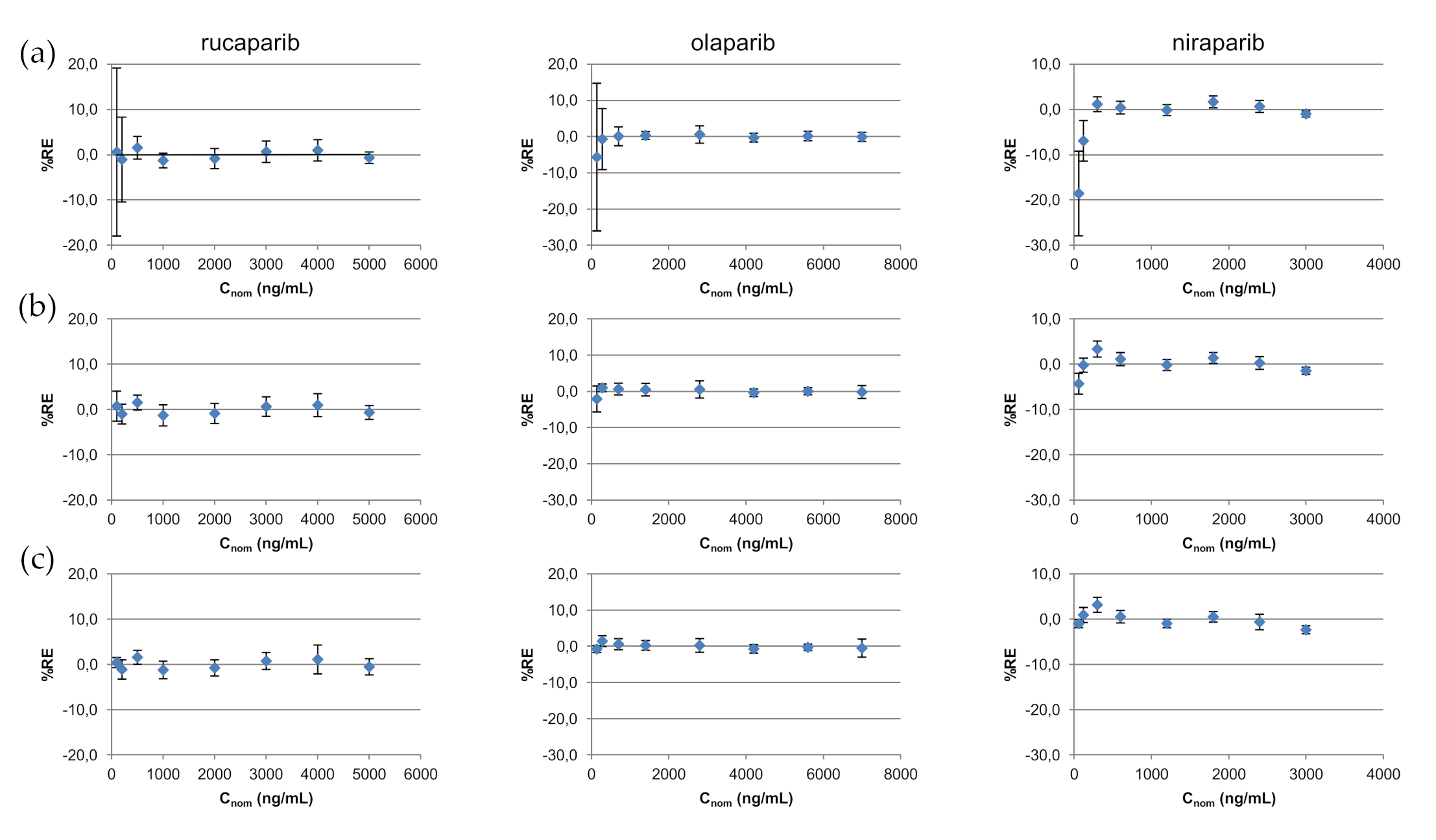

Supplement: Supplementary file 1 [file pharmaceutics-15-01524-s001.zip › Figure S6.tif]
